# 

**Published:** 1821-06-01

**Authors:** 


### Contents

OF

No. 5, Vol. II. for JUNE, 1821.

I. Page

On Affections of the Mucous Membrane of the Digestive Organs; by Drs. Broussais, Abercrombie, Mekel, and Grimaud - - - - - - - - - - - - - - - - 1

II.

Dr. Osborn's Sketch of the Physiology and Pathology of the Urine - - - - - - - - - - - - - - - - - 41

III.

Dr. Conquest's Outlines of Midwifery - - - - - - - - 47

IV.

Messrs. Dalbant, Vaidy, and others, on Arterial Inflammation 50

V.

Mr. Swan, Dr. Kerrison, and Mr. Villers, on Affections of the Nerves - - - - - - - - - - - - - - - 63

VI.

Mr. James Boyle on Indian Cholera - - - - - - - - 85

VII.

Dr. Prout on the Nature and Treatment of Gravel, Calculus, &c. - - - - - - - - - - - - - - - - - 89

VIII.

Mr. Travers on Diseases of the Eye, and their Treatment - 105

IX.

Dr. Whitlock Nicholl's General Elements of Pathology - 133

X.

Dr. Ramsbotham's Practical Observations in Midwifery - - 141

XI.

Dr. Weatherhead on the Waters of Leamington - - - 153

XII.

Mr. Charles Thomas Haden's Practical Observations on the Colchicum Autumnale - - - - - - - - - - 155

XIII.

MILITARY SURGERY.

1. Mr. Guthrie on Gun-Shot Wounds, &c. - - - - - - } 165

2. Dr. Hennen on Military Surgery - - - - - - - - }

3. Messrs. Laurent and Percy on Gun-Shot Wounds - }

XIV.

Mr. H. Sandwith on the Epidemic Fever of Bridlington and Vicinity - - - - - - - - - - - - - - - - - - 203

XV.

Biographical Sketch of the late Dr. James Gregory, of Edinburgh - - - - - - - - - - - - - - - - - 216

XVI.

MISCELLANEOUS INTELLIGENCE.

1. New College in Kentucky - - - - - - - - - - - 219
2. Ventriloquism - - - - - - - - - - - - - - - - *ib.*
3. Preparation of Opium - - - - - - - - - - - - - *ib.*
4. Hunterian Oration - - - - - - - - - - - - - - 220
5. Notice of an Extra Limites Department - - - - - - 221

XVII.

EXTRA LIMITES.

Argus.—No. 1. On Medical Conduct - - - - - - - - 222
Ditto—No. 2. Obstinate Pervigilium - - - - - - - - 224
Ditto—No. 3. The Surreptitious Knight; or, Sir Charlatan Glanderman - - - - - - - - - - - - - - - } 224
Correspondence—Junius—Philo-Criticus—Scriblomania - 228
Bibliographical Record - - - - - - - - - - - - - 230
Notices, &c. &c. &c.- - - - - - - - - - - - - - - 235
Additional Subscribers - - - - - - - - - - - - - 236

